# Electroless Platinum Deposition Using Co^3+^/Co^2+^ Redox Couple as a Reducing Agent

**DOI:** 10.3390/ma14081893

**Published:** 2021-04-10

**Authors:** Loreta Tamasauskaite-Tamasiunaite, Yezdi Dordi, Eugenijus Norkus, Ina Stankeviciene, Aldona Jagminiene, Arnas Naujokaitis, Liudas Tumonis, Vytenis Buzas, Laurynas Maciulis

**Affiliations:** 1 Center for Physical Sciences and Technology, Saulėtekio Ave. 3, LT-10257 Vilnius, Lithuania; eugenijus.norkus@ftmc.lt (E.N.); ina.stankeviciene@ftmc.lt (I.S.); aldona.jagminiene@ftmc.lt (A.J.); arnas.naujokaitis@ftmc.lt (A.N.); 2 Lam Research Corporation, Fremont, CA 94538, USA; yezdi.dordi@lamresearch.com; 3 NanoAvionics, JSC, Mokslininkų Str. 2A, LT-08412 Vilnius, Lithuania; liudas.tumonis@gmail.com (L.T.); vytenis@nanoavionics.com (V.B.); laurynas.maciulis@vgtu.lt (L.M.)

**Keywords:** platinum, cobalt, electroless deposition, EQCM

## Abstract

In the present work, the kinetics of electroless deposition of Pt, using a cobalt ion redox system (Co^3+^/Co^2+^) as a reducing agent, has been investigated. The deposition rate of Pt depends on the pH, concentration of reactants, and temperature. The deaeration and bubbling of the plating solution with argon play an essential role. It was found that 0.11 mg cm^−2^ of Pt films could be deposited on the surface of a roughed glass sheet in one hour without replenishing the solution. Additional data have been obtained on the grounds of electrochemical quartz crystal microbalance experiments. The bubbling (agitation) of the electroless Pt plating solution with argon during the deposition of Pt results in a higher deposition rate and is ca. 3 µg cm^−2^ min^−1^. The Pt deposition rate is far less, and is as low as 0.14 µg cm^−2^ min^−1^ when the electroless Pt plating solution is not bubbled with argon during the deposition of Pt.

## 1. Introduction

Nowadays, electroless metal plating processes are applied in many areas of research and industry, e.g., in metallization, galvanoplastic, microcircuits, and optoelectronics. Moreover, they are successfully used to form catalysts for fuel cells and in various catalytic processes, such as catalytic steam methane reforming (SMR), methanol oxidation, etc. [1,2,3,4,5,6,7,8,9]. In general, the mechanism of electroless metal deposition (Equation (1)) is considered as the coupling of the cathodic reaction of reducing of metal ions (Equation (2)) and the anodic reaction of oxidation of the reducing agent (Equation (3)), occurring simultaneously at the surface to be plated:

(1) $${\mathrm{MeL}}_{m}^{n+}+\mathrm{Red}\to \mathrm{Me}+\mathrm{mL}+{\mathrm{Ox}}^{n+},$$

(2) $${\mathrm{MeL}}_{m}^{n+}+{\mathrm{ne}}^{-}\to \mathrm{Me}+\mathrm{mL},$$

Red → Ox^n+^ + ne^−^, (3)

Therefore, under open-circuit conditions, an electrode attains a mixed potential (*E*_m_) due to both partial reactions (Equations (2) and (3)) occurring at equal rates [10,11,12,13].

A sufficiently strong reducing agent is required for autocatalytic metal deposition. The use of traditional reducing agents, such as borohydride, borane dimethylamine, and hypophosphite, results in the deposition of non-pure metal coatings that contained boron or phosphorous [14,15,16]. Moreover, when using hydrogen-containing reducing agents, the deposited coating structure has large defects due to the evolution of hydrogen gas. The use of hydrogen-containing reducing agents is also connected with environmental and technological problems: (1) the plating bath cannot be recycled, i.e., the reducing agent oxidizes irreversibly; (2) formaldehyde and most ligands are environmentally unacceptable; and (3) the plating rate and solution stability are not high enough. The search for new types of solutions, which would be more environmentally-friendly and have a higher plating rate and solution stability, has been made in some works [17,18]. For the reasons mentioned above, the search for and investigations of new reducing agents, e.g., charge-transfer reducers, *viz.* the different oxidation state metal-ion redox couples, are ongoing. In this case, multivalent metal ions with lower oxidation states are strong enough to reduce other metal ions to metallic states: Cr^2+^, Cr^3+^, Ti^2+^, Ti^3+^, V^2+^, V^3+^, V^4+^, Cu^+^, Sn^2+^, Fe^2+^. Generally, the most pronounced catalytic effect has been observed for the Co^3+^/Co^2+^ redox couple. For the first time, the use of Co^2+^ complexes with ethylenediamine as reducing agents for electroless copper deposition was documented by Vaskelis with co-workers in 1995 [19]. The authors carried out detailed investigations on the behavior of the Co^3+^-Co^2+^-ethylenediamine redox couple in systems related to electroless copper deposition [20,21,22,23,24,25,26,27,28]. Ethylenediamine as a ligand is not an exclusive amine for Co^3+^-Co^2+^ redox couples in the electroless copper plating systems. Co^2+^ complexes with other higher polyamines, e.g., propylene diamine (propane-1,2-diamine) [29,30], diethylenetriamine [31,32], or pentaethylenehexamine [33], and are eligible reducing agents to reduce Cu^2+^ to the metallic state on a surface to be plated.

It is worth noting that Co^2+^ complexes with different amines have found application as reducing agents for electroless deposition of metals different from Cu. The authors successfully used the Co^3+^-Co^2+^-ammonia redox couple for the deposition of silver coatings [34,35]. Electroless gold plating was carried out when using trimethylene diamine as a Co^2+^ ligand [29]. Electroless deposition of platinum using Co^2+^ complexes with diethylenetriamine as a reducing agent was documented recently [36].

In this work, we investigated autocatalytic reduction of Pt^4+^ by the Co^3+^-Co^2+^-diethylenetriamine redox couple. The Co^2+^ metal ion reducing agent-containing bath is operable below room temperature and with a low pH. Additionally, the kinetics of electroless deposition of platinum have been investigated using electrochemical quartz crystal microgravimetry (EQCM). The method is based on the Sauerbrey’s equation [37], where the measured frequency changes of the quartz crystal are correlated with the mass changes according to Equation (4):

(4) $$\Delta f=-2\frac{f_{0}^{2}\Delta m}{S\sqrt{\mu_{q}\rho_{q}}}$$

where *f*_0_—is the resonant frequency of the quartz crystal, *S* is the piezoelectrically active area (cm^2^), *μ_q_* is the shear modulus of the quartz (2.947 ⋅ 10^11^ g cm^−1^ s^−2^) and *ρ_q_* is its density (2.648 g cm^−3^) [38]. As seen from Equation (5),

Δ*m* = −Δ*fSC*_q_ (5)

where *C*_q_—the quartz crystal sensitivity constant. For a 6 MHz quartz crystal, it is 12.26 ng cm^−2^ Hz^−1^. This sensitive method allows determining small in situ changes in the electrode mass, which are directly proportional to the changes in the quartz crystal resonant frequency.

## 2. Materials and Methods

**Figure 1:**
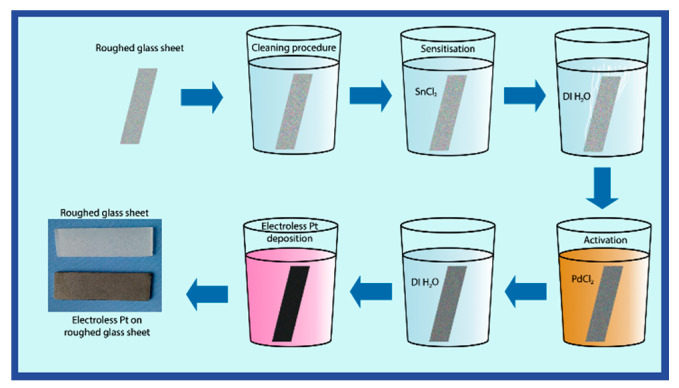
Scheme of electroless platinum deposition.

Electroless Pt films were deposited onto a roughed glass sheet (1 cm ⋅ 2.5 cm) at a temperature of 20 °C. The surface roughness factor of the glass sheet was ca. 10. The scheme of electroless Pt deposition is shown in Figure 1. At first, the roughed glass sheet’s cleaning procedure (the same in all experiments) was carried out by degreasing the glass sheet in a K_2_Cr_2_O_7_ + H_2_SO_4_ solution. After that, the glass sheet was sensitized in a 1 g L^−1^ SnCl_2_ solution for 1 min, rinsed with distilled water, then activated in a 1 g L^−1^ PdCl_2_ solution for 1 min, rinsed with deionized water, and then immersed into the electroless Pt plating bath.

The electroless Pt plating bath containing 0.004–0.012 mol L^−1^ H_2_PtCl_6_, 0.4 mol L^−1^ NH_4_OH, and 0.16 mol L^−1^ diethylenetriamine (dien) was prepared. The addition of HCl adjusted the solution pH to 7.5. It is well-known that the Co^2+^ compounds react with oxygen in alkaline solutions [39]. The plating solution was deaerated with argon (Ar) for 10 min to remove the oxygen. Then, the required amount of CoCl_2_ in the range of 0–0.25 mol L^−1^ was added to the electroless plating solution. Later studies of Pt deposition were carried out in the deaerated solution with continuous Ar bubbling through the solution. The main experiments were performed at a temperature of 20 °C and the time (*t*_dep_) of electroless Pt deposition was 30 min unless otherwise stated.

A SEM/FIB workstation Helios Nanolab 650 (FEI, Eindhoven, The Netherlands) with an energy dispersive X-ray (EDX) spectrometer INCA Energy 350 XMax 20 (Oxford Instruments, Abingdon, UK) was used to investigate the morphology of the Pt films deposited on the surface of a glass sheet.

A tearing fastness test was used to evaluate the adhesion strength of the deposited Pt layer on a roughed glass sheet. Briefly, the tearing fastness test was performed by pasting tape on Pt coating’s surface and then tearing the tape off quickly to observe the surface peeling condition of the Pt layer.

Electrochemical quartz crystal microgravimetry (EQCM) was used to investigate the kinetics of electroless deposition of Pt films. EQCM setup is described in detail in Reference [40].

Before the electroless platinum deposition measurements, a copper layer was electrodeposited on a gold sublayer onto quartz crystals installed at the bottom of the cell from a solution containing 1.0 mol L^−1^ CuSO_4_ and 0.5 mol L^−1^ H_2_SO_4_ at a current of 10 mA for 1 min. Initially, the instantaneous rate of electroless Pt deposition was determined on the electrodeposited copper surface.

For comparison, the EQCM experiments were carried out in two ways: (i) the deposition of Pt was investigated, then the prepared and deaerated Pt plating solution was not bubbled (agitated) with Ar (denoted as “without Ar bubbling”) during the deposition process; and (ii) the electroless Pt plating solution was bubbled with Ar during the deposition process (denoted as “with Ar bubbling”).

## 3. Results

Electroless Pt films were deposited on the surface of a roughed glass sheet using the Co^3+^/Co^2+^ ions couple as a reducing agent and diethylenetriamine as a complexing agent. Generally, the reduction of Pt^4+^ with Co^2+^ in diethylenetriamine solutions occurs as follows:

Pt^4+^ + 4Co^2+^ → Pt^0^ + 4Co^3+^, (6)

and it is the sum of two (anodic and cathodic) partial reactions, simultaneously occurring on the surface to be plated:

Co^2+^ − e^−^ → Co^3+^, (7)

Pt^4+^ + 4e^−^ → Pt^0^, (8)

**Figure 2:**
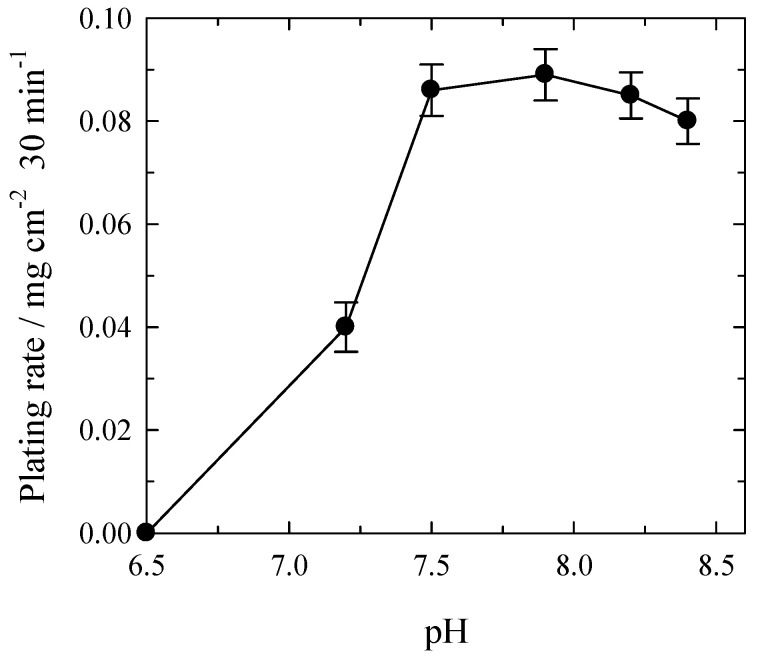
Dependence of plating rate of Pt on the solution pH under conditions with Ar bubbling. Solution contained (mol L^−1^): H_2_PtCl_6_—0.004; NH_4_OH—0.4; complexing agent—0.16; CoCl_2_—0.2; HCl to pH = 7.5; *t*_dep_ = 30 min; 20 °C.

Figure 2 presents the dependence of Pt deposition rate on solution pH. Formation of Pt coatings begins at a pH over 6.5 (Figure 2). A sharp increase in the amount of deposited Pt is observed when pH rises from 6.5 to 7.5. The maximum plating rate obtained was close to 0.09 mg cm^−2^ for 30 min. With further pH increment, the plating rate remains constant or slightly diminishes (Figure 2). It is worth noting that creating the solutions with a pH higher than 8.5 was impossible due to a precipitate formation.

**Figure 3:**
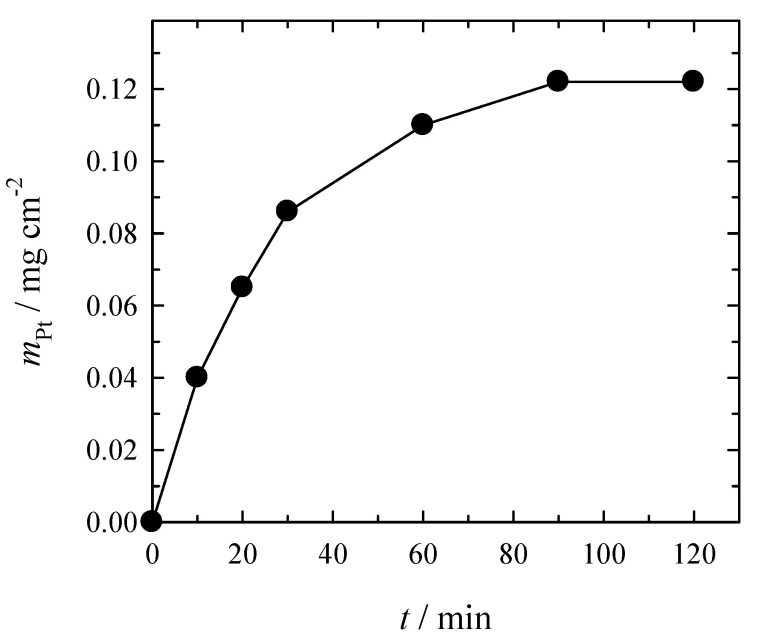
Dependence of deposited Pt mass on the roughed glass sheet on time under conditions with Ar bubbling. The solution composition is the same as in Figure 2.

The dependence of deposited Pt mass on a roughed glass sheet plating time is shown in Figure 3. As evident, the mass of deposited Pt film increases with time. During the first 30 min, the observed deposition rate is the highest, whereas later, it slows down and stops after 2 h (Figure 3). It should be noted that Pt deposits of 0.12 mg cm^−2^ during 90 min can be obtained from the solutions investigated without additional replenishment of the reactants (Figure 3). The decrease in Pt deposition rate can be explained by the formation, accumulation, and adsorption of the reaction products (e.g., Co^3+^-dien complexes) on the surface to be plated, which diminishes the Pt catalytic surface. The same phenomenon was observed in electroless copper plating using Co^3+^/Co^2+^-ethylenediamine complexes as a reducing agent [26]. The authors investigated the copper deposition under unstirred and hydrodynamic conditions. As the jet of electrolyte removes inhibiting Co^3+^ species from the surface and ensures the transport of both reacting species (Co^2+^ and Cu^2+^) to the electrode, the copper deposition rate was ca. 10 times higher compared with that under stationary conditions.

**Figure 4:**
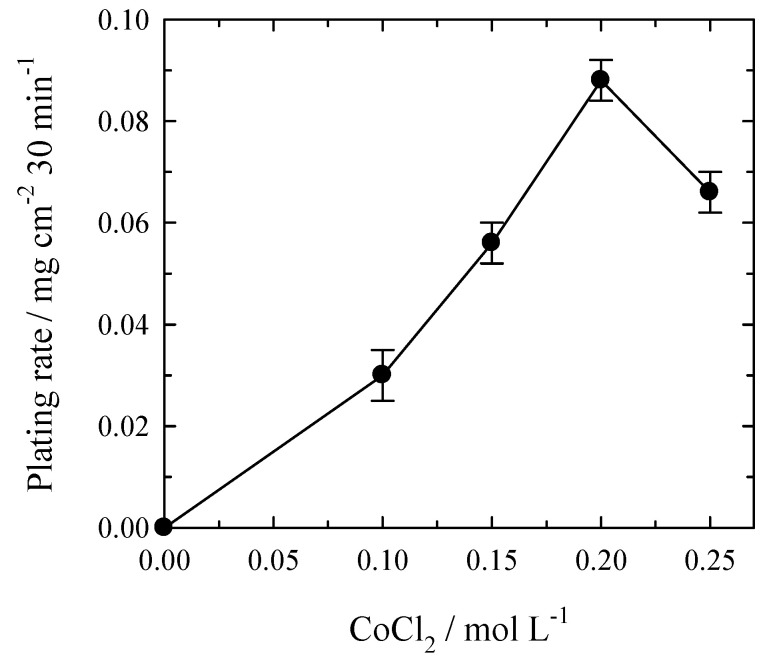
Effect of Co^2+^ concentration on the rate of electroless Pt plating under deaeration with Ar. Solution composition (mol L^−1^): H_2_PtCl_6_—0.004; NH_4_OH—0.4; complexing agent—0.16; HCl to pH = 7.5; *t*_dep_ = 30 min; 20 °C.

At a constant Pt^4+^ concentration, the platinum plating rate increases with the rise in Co^2+^ concentration (Figure 4). The concentration dependence has a distinctly expressed maximum at 0.2 mol L^−1^ of Co^2+^, and the highest Pt deposition rate was found to be ca. 0.09 mg cm^−2^ during 30 min and the thickness being ca. 0.05 µm. A further increase in Co^2+^ concentration results in the slowdown of the platinum plating rate.

**Figure 5:**
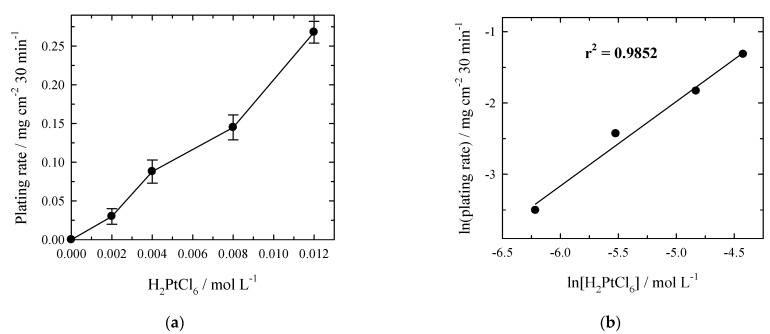
(**a**) Effect of Pt^4+^ concentration on the rate of electroless Pt plating under conditions with Ar bubbling. Solution composition (mol L^−1^): CoCl_2_—0.2; NH_4_OH—0.4; complexing agent—0.16; HCl to pH = 7.5; *t*_dep_ = 30 min; 20 °C. (**b**) Dependence of natural logarithmic plating rates of Pt vs. natural logarithmic bulk concentrations of H_2_PtCl_6_.

Under conditions of constant Co^2+^ concentration, the mass of deposited platinum depends practically linearly on the concentration of Pt^4+^ in the concentration range investigated (Figure 5a). In Figure 5b, natural logarithmic plating rates of Pt vs. natural logarithmic bulk concentrations of H_2_PtCl_6_ were plotted. The slope of the straight line is 0.9852, indicating 1st order kinetics. The maximum electroless platinum deposition rate is ca. 0.27 mg cm^−2^ during 30 min and is observed at a concentration of Pt^4+^ equal to 0.012 mol L^−1^ (Figure 5a). The thickness of the deposited Pt layer was ca. 0.13 µm.

**Figure 6:**
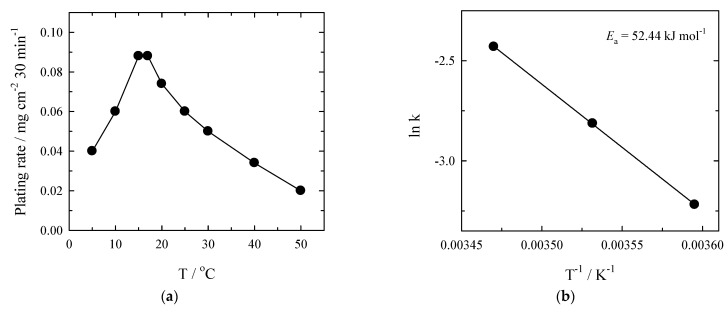
(**a**) Effect of temperature on the rate of electroless Pt plating under conditions with Ar bubbling. The solution composition is the same as in Figure 2. (**b**) The Arrhenius plot calculated from Pt deposition rates for the first three points in the same solution.

The electroless Pt deposition begins at a relatively low temperature (5 °C) and increases up to a distinct maximum value of the temperature, equal to 15–17 °C (Figure 6a). After the increase in temperature from 17 to 50 °C, the platinum plating rate unexpectedly decreases more than fivefold. Interestingly, the platinum deposition rate at a temperature of 50 °C is twice as low as that at 5 °C (Figure 6a). The decrease in Pt plating rate at temperatures higher than 15 °C may be attributed to the changes in solution equilibria at higher temperatures or/and the surface contamination by reaction products formed at higher temperatures. The Arrhenius plot was calculated from the first three points and given in Figure 6b. The activation energy is ca. 52 kJ mol^−1^.

Therefore, based on the data obtained, we can conclude that the optimum operating conditions (high enough plating rate and moderate temperature) are as follows (mol L^−1^): H_2_PtCl_6_—0.004; CoCl_2_—0.2; NH_4_OH—0.4; complexing agent—0.16; pH = 7.5, the temperature being 17–20 °C. Experiments showed that the Pt films with a thickness greater than 0.1 mg cm^−2^ could be obtained on the surface of the glass sheet without replenishment of the solution (Figure 3). The solutions were stable during the electroless Pt deposition, and the reduction of Pt^4+^ occurred only on the surface plated, no considerable signs of the reduction of Pt^4+^ in the solution bulk were observed.

**Figure 7:**
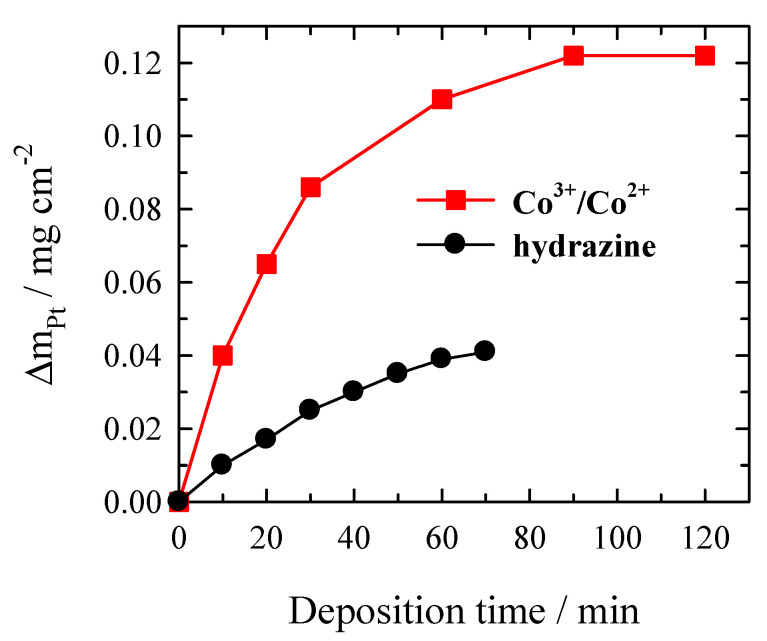
The rate of Pt deposition using different reducing agents.

For comparison, the Pt coating was deposited on the roughed glass sheet using the conventional electroless Pt plating bath described in Reference [1]. The bath contained 0.03 M Na_2_Pt(OH)_6_, 0.12 M ethylenediamine, 0.125 M NaOH, and 0.02 M N_2_H_4_. The solution pH was ~10, the operating temperature was 35 °C. Figure 7 presents the rate of Pt deposition using the Co^3+^/Co^2+^ redox couple and N_2_H_4_ as reducing agents. In the case of Pt plating bath with N_2_H_4_, the reduction of Pt^4+^ occurs in the solution bulk after one h of deposition, indicating the plating solution instability. Comparing the electroless Pt plating process using the Co^3+^/Co^2+^ redox couple and N_2_H_4_ as reducing agents shows the advantages of Co^2+^ complexes as reducing agents. The rate of Pt plating using the Co^3+^/Co^2+^ redox couple as a reducing agent is ca. 3 times higher than that using N_2_H_4_ as a reducing agent. Furthermore, the solution stability of Pt plating bath with Co^2+^ complexes is much better than that with N_2_H_4_ (Figure 7).

**Figure 8:**
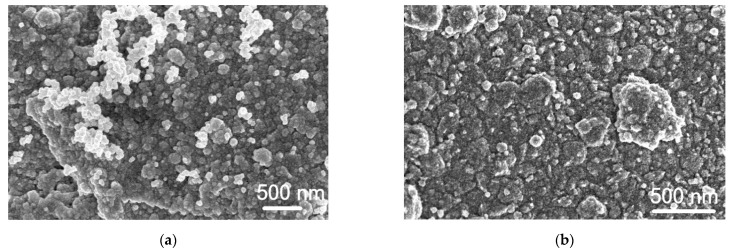
SEM images of the roughed glass sheet after the electroless Pt plating using the Co^3+^/Co^2+^ redox couple (**a**) and hydrazine (**b**) as reducing agents.

Figure 8 presents the electrolessly deposited Pt coatings SEM views, obtained using the Co^2+^ complexes (a) and hydrazine (b) as reducing agents. It is seen that in the case of hydrazine, the coating is built from much larger Pt conglomerates, comparing with that using the Co^2+^ complexes as a reducing agent (Figure 8). As seen, the Pt film obtained is compact and of good quality (Figure 8a). Concerning the adhesion of Pt coatings received using Co^3+^/Co^2+^ and N_2_H_4_ as reducing agents, it can be noted that the adhesion of the coating is higher enough. The tearing test showed no surface peeling for the deposited Pt coatings, indicating a strong adhesion of Pt coatings with a roughed glass sheet.

**Figure 9:**
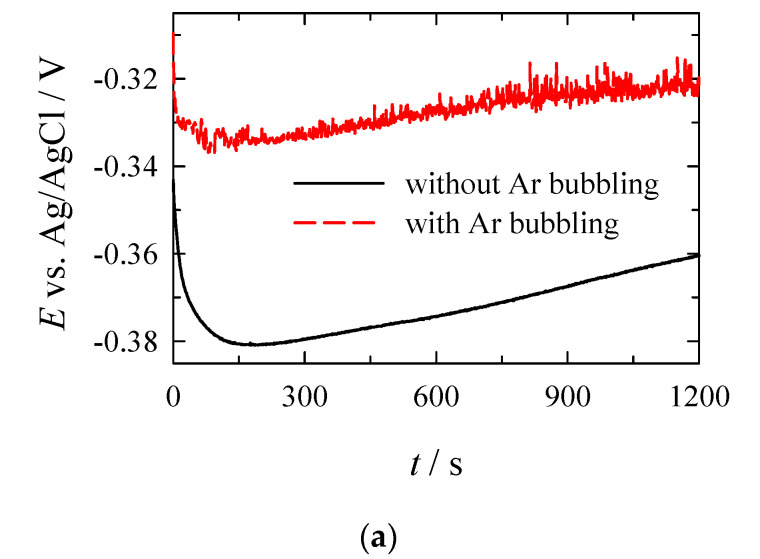

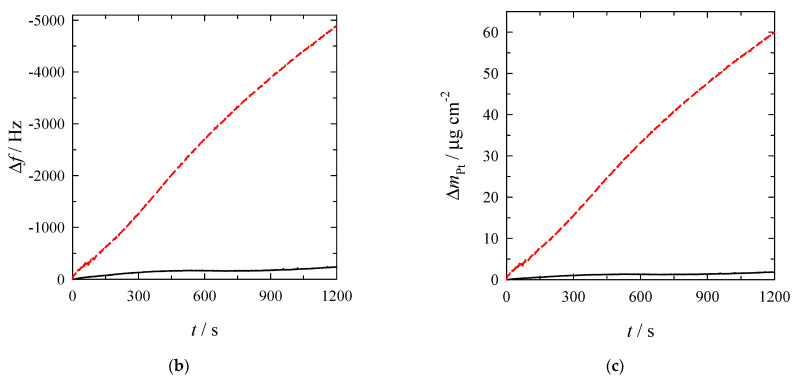
Kinetics of electroless platinum plating under conditions with Ar bubbling (dashed line) and deaerated and not-agitated solution (solid line) under stationary conditions: open-circuit potential (**a**), change in frequency (**b**) and platinum mass gain (**c**). Solution contained (mol L^−1^): H_2_PtCl_6_—0.004; NH_4_OH—0.4; complexing agent—0.16; CoCl_2_—0.2; HCl to pH = 7.5; 20 °C.

The electroless deposition of Pt was investigated in more detail employing electrochemical quartz crystal microbalance. The instantaneous rate of Pt deposition on the initial electroplated copper surface was investigated in solutions, which were deaerated with Ar before the measurements, and during the deposition of Pt, those solutions were not bubbled with Ar, and in solutions under conditions of constant Ar bubbling during the deposition process. The EQCM data on the electroless Pt deposition using the Co^3+^/Co^2+^ redox couple as a reducing agent are shown in Figure 9, which presents the main measured parameters of the electroless Pt deposition: open-circuit potential (a), change in frequency (b), and Pt mass gain (c).

It is evident that in the absence of an external current, the electrode attains a mixed potential (*E*_m_) value (Figure 9a). The open-circuit potential of Cu in the course of electroless deposition is quite stable during the electroless Pt deposition under both conditions, while its values after ca. 150 s slightly shift to more positive values (Figure 9a). During the electroless Pt deposition, the frequency begins to decrease, i.e., the coating mass increases linearly with time (Figure 9b,c). Moreover, the bubbling of the electroless Pt plating solution with argon results in a higher Pt deposition rate. The rate of the electroless Pt deposition under bubbling with argon is ca. 3 µg cm^−2^ min^−1^, whereas the value of plating rate of 0.14 µg cm^−2^ min^−1^ was determined in the solution that was not bubbled during deposition. Notably, under conditions of bubbling with Ar, the Pt films with a thickness greater than 60 µg cm^−2^ may be obtained without replenishing the solution over 20 min (Figure 8c).

## 4. Conclusions

The kinetics of electroless deposition of Pt on a roughed glass sheet, using the cobalt ion redox system (Co^3+^/Co^2+^) as a reducing agent, has been investigated. It has been determined that the deposition of Pt depends on pH, the concentration of reactants, temperature, and the agitation of the plating solution by bubbling with Ar. It was found that the Pt films with a thickness greater than 0.11 mg cm^−2^ could be obtained on the surface of the roughed glass sheet without replenishment of the solution over one h.

The electroless deposition of Pt on the Cu electrode has been investigated using electrochemical quartz crystal microbalance. The agitation of the electroless Pt plating solution by bubbling with argon results in higher deposition rates of Pt—the rate of the electroless Pt deposition is ca. 3 µg cm^−2^ min^−1^. In the case of the non-agitated plating solution, the rate of Pt deposition is significantly lower, e.g., 0.14 µg cm^−2^ min^−1^. The Pt films obtained are compact and of good quality.
